# Evaluation of an Advanced Nine-Parameter Cytomorphological Grading System for Improving Preoperative Accuracy and Histopathological Correlation in Breast Carcinoma

**DOI:** 10.7759/cureus.117997

**Published:** 2026-10-15

**Authors:** Shrikant Nema, Santosh Jayant, Abhiraj Ramchandani, Mamta Gupta, Mahendra Singh Hora, Amarnath Yadav, Pawan Bhambani

**Affiliations:** 1 Pathology and Laboratory Medicine, Amaltas University, Dewas, IND; 2 Pathology and Laboratory Medicine, Index Medical College, Hospital & Research Centre, Indore, IND

**Keywords:** bloom-richardson histological grade, breast carcinoma, cyto-histological correlation, cytological grading, diagnostic specificity, fine-needle aspiration cytology (fnac), iac yokohama system, linear weighted kappa, robinson grading system

## Abstract

Introduction: Preoperative cytological grading of breast carcinoma plays an indispensable role in guiding clinical staging, surgical management, and neoadjuvant therapy. While standardized classifications like the National Cancer Institute Bethesda guidelines and the International Academy of Cytology (IAC) Yokohama system provide consistent five-tier reporting, diagnostic gray zones persist, and established six-parameter frameworks (e.g., Robinson's criteria) often lack the biological nuance required to evaluate borderline and high-grade tumors. The primary objective of this prospective study was to evaluate an improvised nine-parameter cytomorphological grading framework (scores 9 to 27) applied specifically to invasive breast carcinomas and assess its agreement against postoperative modified Bloom-Richardson histological grades in patients with available surgical specimens, while secondarily comparing performance directly against Robinson's system.

Materials and methods: Fine-needle aspiration cytology (FNAC) was performed on 106 female patients presenting with palpable breast lumps between July 15, 2024, and July 14, 2026. Cytological smears were stained with Papanicolaou and Leishman-Giemsa stains. Smears fulfilling the adequacy requirement of at least six distinct epithelial clusters were classified into five tiers. Confirmed malignant aspirates were graded using an improvised nine-parameter scoring rubric (scores 9 to 27) structured into distinct, non-overlapping tiers: Grade I (9-13), Grade II (14-20), and Grade III (21-27), evaluating epithelial and myoepithelial distribution, cluster cohesiveness, and margin irregularities along with nuclear characteristics. Findings were statistically correlated against histopathological grades in 50 surgical mastectomy specimens. Inter-method agreement was evaluated using linear weighted Cohen's kappa (kappa w) with standard error (SE) and 95% confidence intervals (CI).

Results: The cohort was categorized into 52 benign cases, four atypical/suspicious cases, and 50 malignant cases. The nine-parameter system classified malignant lesions into 11 Grade I cases, 31 Grade II cases, and eight Grade III cases. Concordance with modified Bloom-Richardson histological grades was statistically significant (chi square = 238.2, p < 0.001), yielding an overall concordance of 92.0% (46/50), a linear weighted kappa (kappa w) of 0.908 (95% CI: 0.812-1.000; SE = 0.049), and an unweighted kappa (kappa) of 0.865 (95% CI: 0.741-0.989), with a 100% concordance rate for Grade III lesions. In comparison, Robinson’s criteria evaluated across the same 50 cases demonstrated 78.0% concordance (39/50) with kappa w = 0.694 (95% CI: 0.528-0.860).

Conclusions: The improvised nine-parameter cytomorphological system demonstrated strong concordance with histological tumor grade in this single-center cohort, showing improved stratification of intermediate-grade lesions compared with Robinson's criteria. Because evaluation was restricted to 50 surgically treated patients without concurrent biomarker assessment (ER, PR, HER2, and Ki-67) or formal multi-observer reproducibility testing, findings should be regarded as preliminary, and multi-center external validation is necessary before recommending routine clinical implementation.

## Introduction

In modern clinical oncology, the management of palpable breast masses relies extensively on the "triple assessment" approach comprising clinical palpation, radiological imaging, and pathological evaluation [1]. Global epidemiological surveillance indicates that breast cancer is the leading cause of cancer morbidity and mortality among women worldwide, with developing nations such as India bearing a substantial clinical burden exceeding 200,000 newly diagnosed cases each year [2,3]. In healthcare settings where core needle biopsy (CNB) is constrained by technical logistics, turnaround time, or financial considerations, fine-needle aspiration cytology (FNAC) remains an indispensable primary diagnostic tool [4].

The standardization of breast cytopathology reporting progressed notably from the 1996 National Cancer Institute (NCI) Bethesda recommendations to the 2016 International Academy of Cytology (IAC) Yokohama framework, which codified a five-tier risk stratification architecture [5,6]. Nonetheless, reliable cytological grading remains critical to bridge preoperative evaluation and definitive therapeutic strategy [7,8]. While Robinson's classic six-parameter system has provided a dependable baseline, its diagnostic granularity is often insufficient to resolve borderline atypical lesions or mirror the aggressiveness of modern histological subtypes [9].

The primary objective of this prospective study was to evaluate an improvised nine-parameter cytomorphological grading framework (scores 9 to 27) applied specifically to invasive breast carcinomas and assess its agreement against postoperative modified Bloom-Richardson histological grades in patients with available surgical specimens (n = 50). The secondary objectives were to describe the score distribution across the 106-patient cohort and conduct an exploratory head-to-head comparison between this nine-parameter system and Robinson's classical schema using linear weighted Kappa statistics, 95% confidence intervals (CI), and percentage concordance in the same histologically verified surgical cohort [10,11].

## Materials and methods

This prospective diagnostic correlation study was conducted in the Department of Pathology at Amaltas Institute of Medical Sciences, Amaltas University, Dewas, Madhya Pradesh, India. The study spanned a two-year recruitment and follow-up period from July 15, 2024, to July 14, 2026. A total of 106 female patients aged 28 to 75 years (mean age: 48.41 years) with clinically palpable breast lesions were included consecutively [11].

Formal ethical clearance was obtained from the Institutional Ethics Committee (IEC) of Amaltas Institute of Medical Sciences, Dewas (Protocol Approval Letter Reg. No.: EC/NEW/INST/2022/2692; CDSCO Reg. No.: ECR/1145/Inst/MP/2018/RR-22) following review and approval during the committee meeting held on July 2, 2024. Written informed consent was secured from all participants.

The diagnostic and grading classifications applied-including the NCI Bethesda system [12], the IAC Yokohama system [13], Robinson's cytological grading system [14], and the Elston-Ellis modified Bloom-Richardson histological grading schema [15]-are standard, public-domain academic medical tools. No commercial licensing agreements or third-party permissions were required for their clinical and research application. All foundational sources have been cited with primary resolving DOIs/URLs.

Aspirations were carried out by experienced pathologists utilizing 20 mL sterile disposable syringes with 20-22-gauge needles under aseptic conditions without local anesthesia. Smears were fixed in 95% ethyl alcohol for Papanicolaou staining and air-dried for Leishman-Giemsa staining. Minimum specimen adequacy was defined as the presence of six or more viable, well-visualized epithelial cell clusters [16].

Evaluation followed a structured two-step protocol.

Step 1 (risk triage)

All 106 aspirates were initially screened into five general categories: benign, atypical, suspicious, malignant, and unsatisfactory [5,6]. Lesions categorized as benign (52 cases) and atypical/suspicious (four cases) were managed clinically under triple assessment and were not assigned a numerical malignant grade.

Step 2 (advanced nine-parameter grading)

Lesions designated as definitive malignant invasive carcinomas (n = 50) underwent advanced cytomorphological assessment across nine specific parameters: cohesiveness of epithelial clusters, cluster margins, distribution of epithelial and myoepithelial cells, cell size, cell uniformity, nuclear:cytoplasmic (N:C) ratio, nuclear margin regularity, chromatin texture, and nucleolar prominence. Each parameter was scored on an ordinal scale of 1 to 3, yielding a cumulative score strictly between 9 and 27 (Table 1).

**Table 1:** Criteria and scoring rubric for the improvised nine-parameter cytomorphological grading system. Source: designed and formulated by the authors of the present study, adapted and expanded from Robinson et al. [8] and the architectural discohesion criteria of Yu et al. [17]. Note on application and cutoffs: this numerical scoring rubric applies strictly to Category 5/malignant aspirates, with total cumulative scores ranging from 9 to 27. Non-overlapping cutoffs: Grade I: 9-13; Grade II: 14-20; Grade III: 21-27. Non-malignant aspirates are categorized via initial diagnostic triage and are not scored using this numerical rubric (removing the former reference to “benign: score ≤ 5”). N:C: nuclear:cytoplasmic

Cytomorphological parameter	Score 1	Score 2	Score 3
Cell cluster cohesiveness	Closely packed, tight sheets and syncytia	Mildly discohesive; few loose cell clusters	Marked discohesion; predominantly dispersed single epithelial cells
Cell cluster margins	Distinct, smooth, and regular boundaries	Mixed smooth and irregular borders	Ragged, highly irregular, and ill-defined margins
Epithelial cells (EC) & myoepithelial cells (MEC)	Predominantly single bare EC nuclei with streaming bipolar MECs	Mixed pattern; <50% single ECs with scattered bipolar MECs	Abundant discohesive malignant ECs with rare or absent MECs
Cell size	1 to 2 times erythrocyte diameter	3 to 4 times erythrocyte diameter	Greater than 5 times erythrocyte diameter
Cell uniformity	Monomorphic cell population	Mild to moderate cellular pleomorphism	Overt, marked cellular pleomorphism
N:C ratio	Normal to minimal increase	Moderately increased	Markedly elevated
Nuclear margin	Smooth, thin, and continuous	Occasional nuclear membrane folds	Highly irregular with thick folds, notches, and clefts
Nuclear chromatin	Finely granular, evenly distributed, vesicular	Moderately clumped and granular	Coarsely clumped, irregular with parachromatin clearing
Nucleolus	Inconspicuous or absent	Small, occasionally discernible	Large, prominent, pleomorphic, and often multiple

The non-overlapping diagnostic score thresholds were defined as follows: Grade I (well differentiated): score 9-13; Grade II (moderately differentiated): scores 14-20; and Grade III (poorly differentiated): scores 21-27. Smears were evaluated in consensus by cytopathologists who were blinded to subsequent post-surgical histological findings. Formal independent multi-observer blinded scoring rounds and intraobserver repeat-scoring protocols were not conducted as part of this preliminary investigation.

Smears were initially screened into five categories: benign, atypical, suspicious, malignant, and unsatisfactory [5,6]. Lesions designated as suspicious or malignant underwent advanced cytomorphological assessment across nine specific parameters: cohesiveness of epithelial clusters, cluster margins, distribution of epithelial and myoepithelial cells, cell size, cell uniformity, nuclear:cytoplasmic (N:C) ratio, nuclear margin regularity, chromatin texture, and nucleolar prominence. Each parameter was scored from 1 to 3, yielding a cumulative score between 9 and 27, as detailed in Table 1.

Histological evaluation was conducted on formalin-fixed, paraffin-embedded surgical tissue sections obtained from 50 consecutive patients with cytologically confirmed invasive carcinoma who subsequently underwent modified radical mastectomy at our center. The remaining 56 patients did not undergo mastectomy: 52 had benign lesions managed conservatively under triple assessment protocols, and four had atypical/suspicious cytology evaluated by localized excision confirming benign proliferative disease without mastectomy-based grading. Tissue sections were stained with hematoxylin and eosin (H&E) and graded blindly according to the Elston-Ellis Nottingham modification of the Bloom-Richardson grading system, assessing tubule formation, nuclear pleomorphism, and mitotic frequency [10].

Statistical calculations were performed using SPSS version 26.0 (IBM Corp., Armonk, NY, USA). Associations between categorical variables were evaluated using Pearson's chi-squared test. Because histological and cytological grades are ordered categories (ordinal tiers: Grade I, Grade II, and Grade III), inter-method concordance was evaluated using linear weighted Cohen's kappa coefficient (kappa w) alongside standard error (SE) and 95% CI, supplemented by unweighted kappa (kappa). A direct head-to-head comparison with Robinson's six-parameter grading was evaluated across the identical 50 histologically verified cases. A two-tailed p-value < 0.05 was considered statistically significant.

## Results

In the present study, of the total of 106 cases evaluated cytologically, 52 cases (49.06%) demonstrated benign cytomorphology (Figure 1), four cases (3.77%) showed atypical ductal hyperplasia or suspicion for malignancy, and 50 cases were confirmed invasive ductal carcinomas (IDCs).

**Figure 1:**
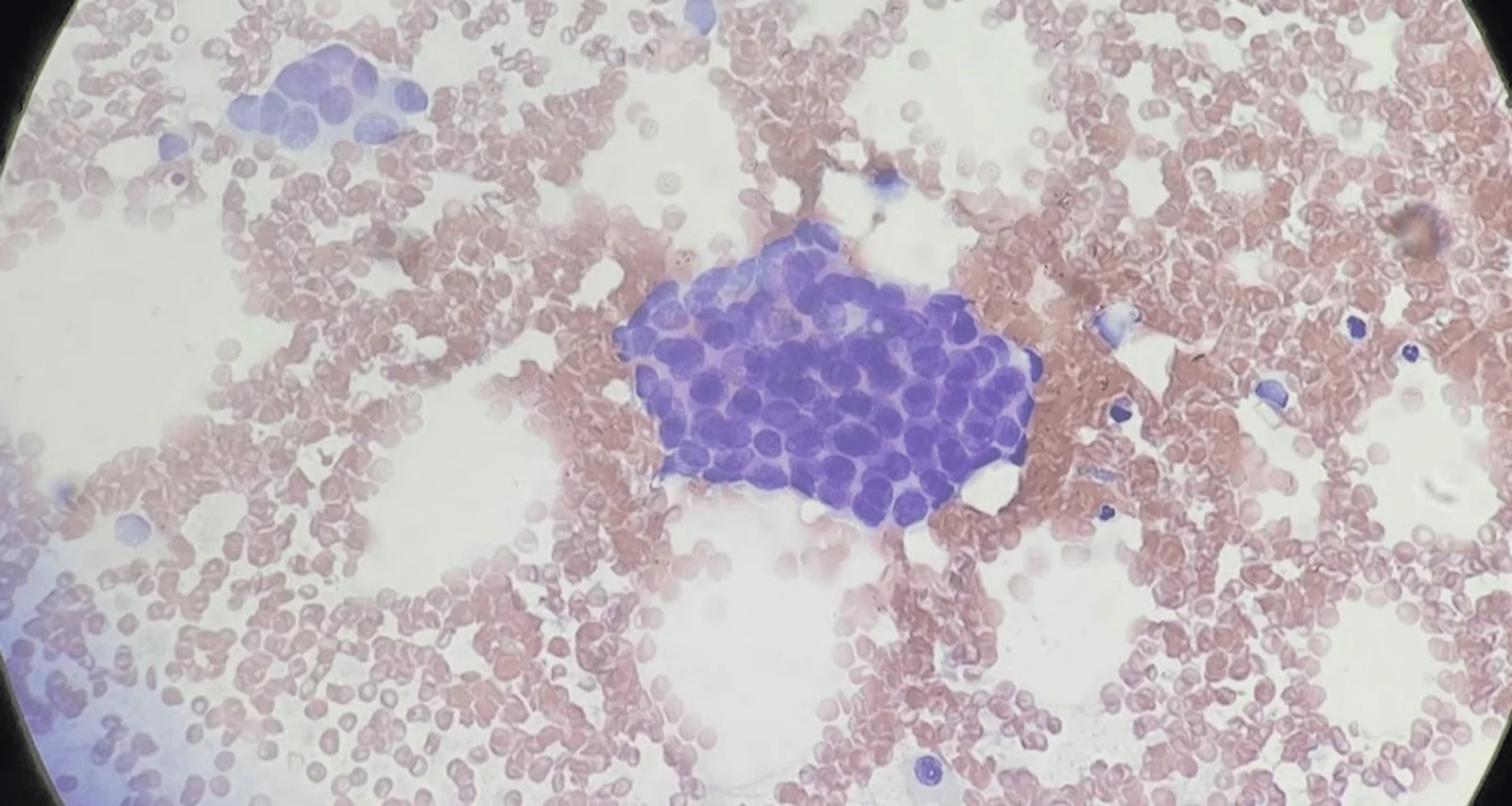
Photomicrograph showing benign ductal epithelial cells arranged in cohesive monolayer sheets with bland nuclear features and background bipolar myoepithelial cells (Leishman-Giemsa, 400×).

Applying the nine-parameter grading system strictly to the 50 malignant cases categorized them into 11 (22.0% of carcinomas; 10.38% of total cohort) as Grade I (scores 9-13) (Figure 2), 31 (62.0% of carcinomas; 29.25% of total cohort) as Grade II (scores 14-20) (Figure 3), and eight (16.0% of carcinomas; 7.55% of total cohort) as Grade III (scores 21-27) (Figure 4), as summarized in Table 2.

**Figure 2:**
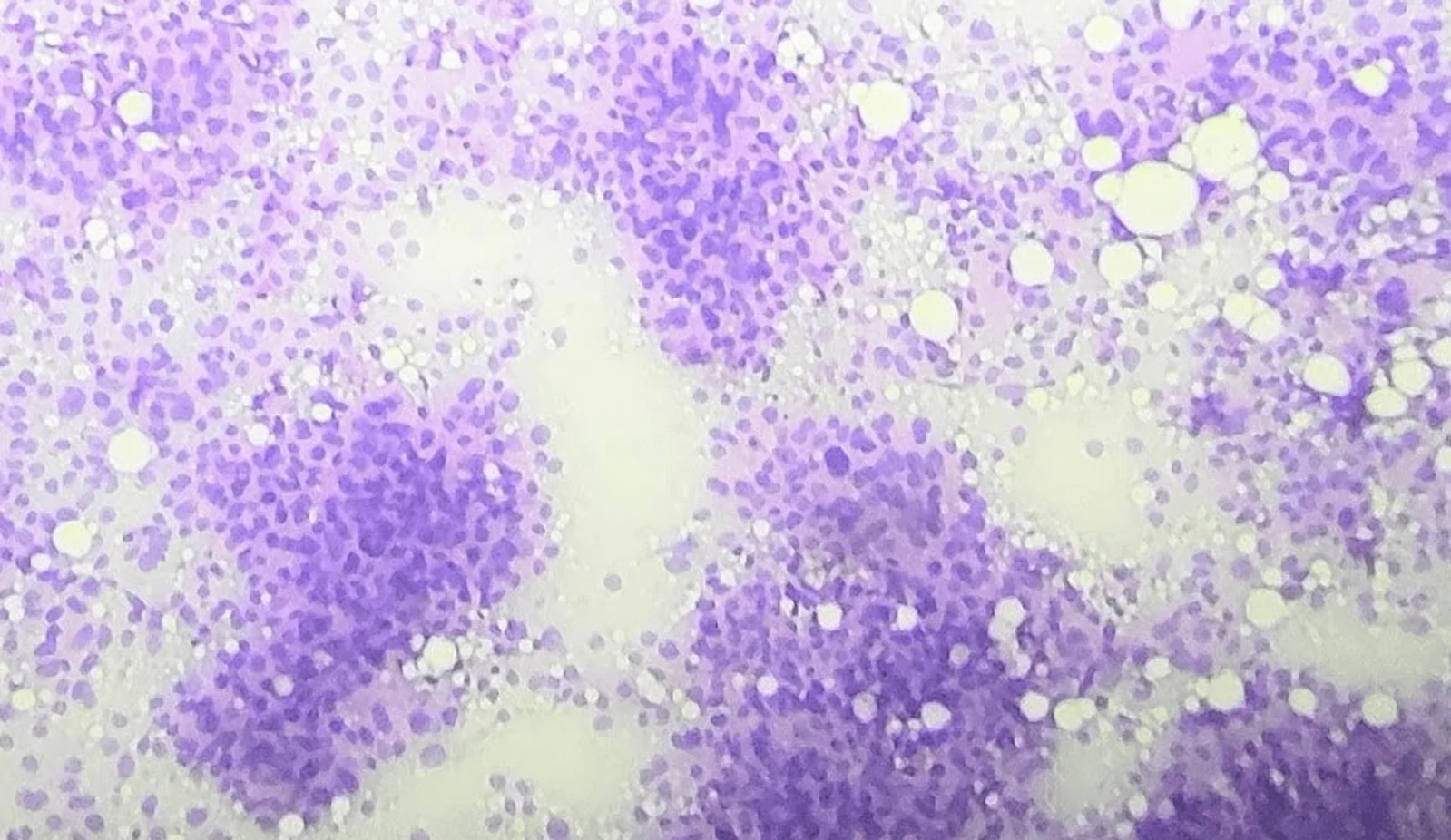
Photomicrograph of Grade I invasive ductal carcinoma demonstrating cohesive tight clusters of monomorphic cells with mild anisokaryosis and reduced myoepithelial cells (Papanicolaou stain, 400×).

**Figure 3:**
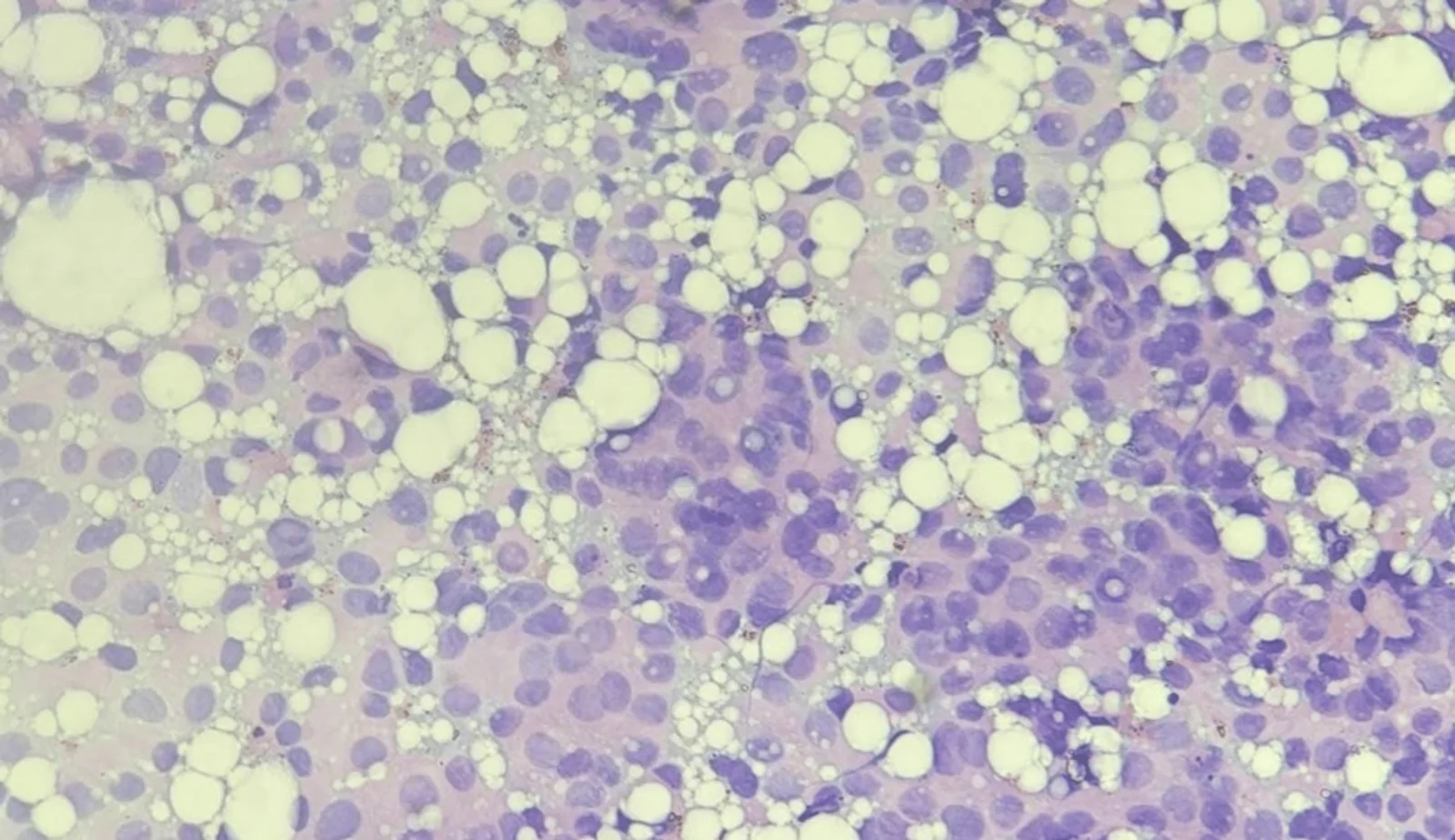
Photomicrograph of Grade II invasive ductal carcinoma displaying loose cell clusters, moderate nuclear pleomorphism, folded nuclear membranes, and granular chromatin (Leishman-Giemsa, 400×).

**Figure 4:**
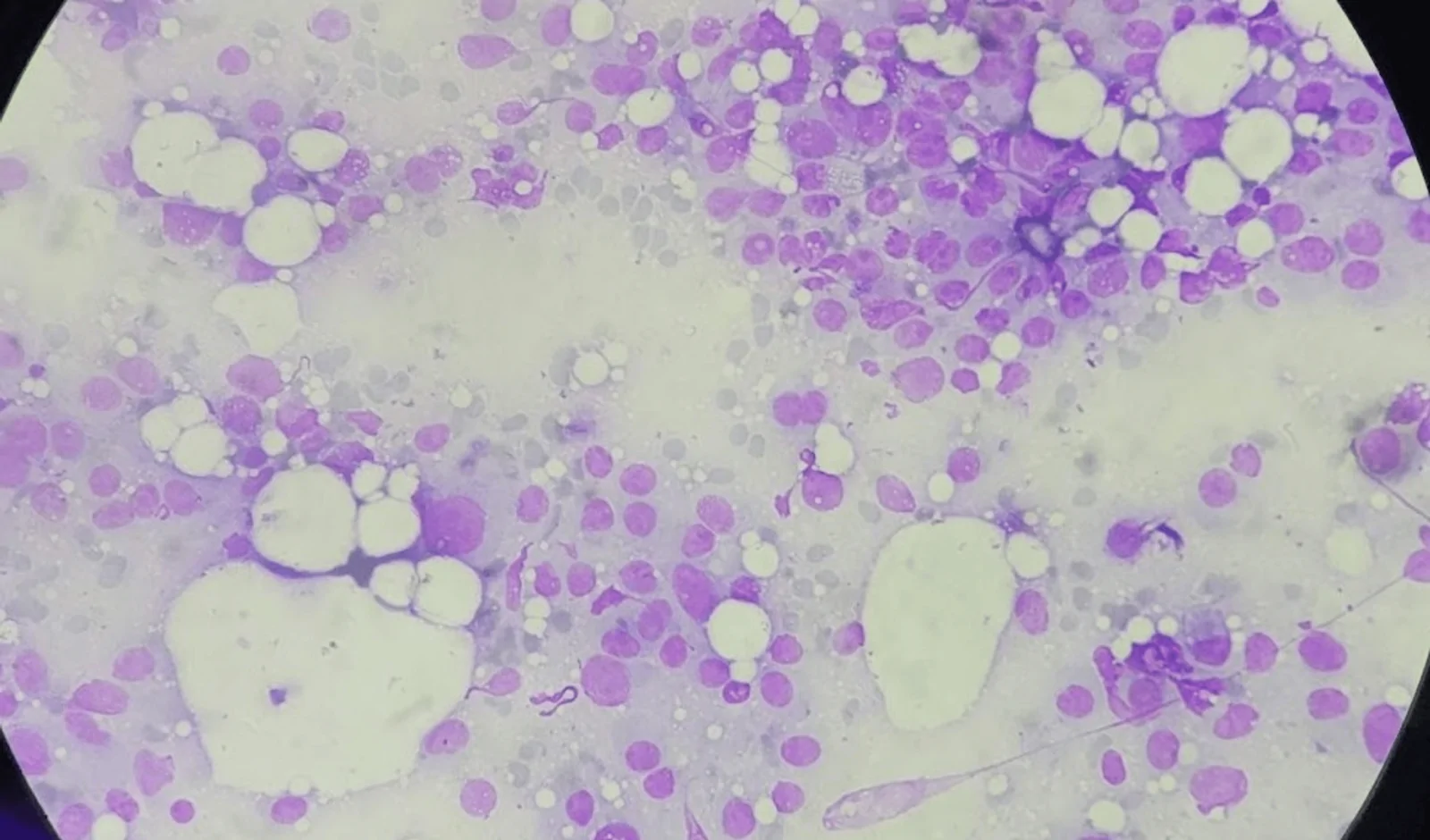
Photomicrograph of Grade III invasive ductal carcinoma depicting complete discohesion, marked cellular and nuclear pleomorphism, high N:C ratio, coarse chromatin clumps, and prominent macronucleoli (Leishman-Giemsa, 400×). N:C: nuclear:cytoplasmic

**Table 2:** Cytomorphological score distribution and category stratification (N = 106). IDC: invasive ductal carcinoma

Diagnostic category	Cytological grade tier	Score range	Number of cases (n)	Percentage (%)
Benign lesions	Non-malignant (benign)	Categorical triage	52	49.06%
Atypical/suspicious	Indeterminate/atypical	Categorical triage	4	3.77%
Invasive carcinoma	Grade I IDC	Scores 9-13	11	10.38%
Invasive carcinoma	Grade II IDC	Scores 14-20	31	29.25%
Invasive carcinoma	Grade III IDC	Scores 21-27	8	7.55%
Total	-	-	106	100.00%

Direct comparison between the nine-parameter grading system and Robinson's established cytological grading framework demonstrated strong statistical association across all 106 cases, as outlined in Table 3 (chi square = 149.39, df = 9, p < 0.001).

**Table 3:** Cross-tabulation and concordance between current nine-parameter grading and Robinson's system (N = 106).

Present system grade	Robinson benign	Robinson Grade I	Robinson Grade II	Robinson Grade III	Total	Statistical comparison
Benign (triage)	52	0	0	0	52	Chi square = 149.39, df = 9, p < 0.001
Atypical/suspicious	4	0	0	0	4	
Grade I (scores 9-13)	0	11	0	0	11	
Grade II (scores 14-20)	0	1	27	3	31	
Grade III (scores 21-27)	0	0	0	8	8	
Total	56	12	27	11	106	

Histopathological correlation was performed on the 50 patients who underwent definitive mastectomy (Table 4). All eight cytological Grade III lesions were confirmed as histological Grade III (100% concordance). Of the 31 cases graded cytologically as Grade II, 27 (87.1%) matched histological Grade II, while one was histological Grade I and three were histological Grade III. All 11 definitive Grade I cytological cases were concordant with histological Grade I.

**Table 4:** Cyto-histological correlation of the improvised nine-parameter system vs. Robinson's system with modified Bloom-Richardson histopathology (n = 50 mastectomy cases). CI: confidence interval

Grading system	Cytological grade	Histological Grade I (n = 12)	Histological Grade II (n = 27)	Histological Grade III (n = 11)	Total (n = 50)	Statistical indices & agreement
Nine-parameter system	Grade I (9-13)	11	0	0	11	Concordance: 92.0% (46/50), linear weighted kappa w = 0.908 (95% CI: 0.812-1.000), unweighted kappa = 0.865 (95% CI: 0.741-0.989), chi square = 238.2, p < 0.001
	Grade II (14-20)	1	27	3	31	
	Grade III (21-27)	0	0	8	8	
	Total	12	27	11	50	
Robinson's system	Grade I (6-11)	9	3	0	12	Concordance: 78.0% (39/50), linear weighted kappa w = 0.694 (95% CI: 0.528-0.860), unweighted kappa = 0.627 (95% CI: 0.449-0.805), chi square = 54.16, p < 0.001
	Grade II (12-14)	3	22	2	27	
	Grade III (15-18)	0	2	9	11	
	Total	12	27	11	50	

Overall concordance between the nine-parameter cytological system and histological grade was 92.0% (46/50). Statistical evaluation demonstrated a linear weighted kappa of kappa w = 0.908 (95% CI: 0.812-1.000; SE = 0.049) and an unweighted kappa of kappa = 0.865 (95% CI: 0.741-0.989) with a chi-squared statistic of 238.2 (p < 0.001).

In contrast, when Robinson's six-parameter grading was correlated with histological grade on the identical 50 cases, concordance was 78.0% (39/50), yielding a linear weighted kappa of kappa w = 0.694 (95% CI: 0.528-0.860; SE = 0.085) and an unweighted kappa of kappa = 0.627 (95% CI: 0.449-0.805; chi square = 54.16, p < 0.001). The nine-parameter framework resolved four discordant intermediate cases.

## Discussion

Preoperative evaluation of palpable breast masses by FNAC is a well-established diagnostic technique praised for its rapid turnaround time, cost-efficiency, and high patient acceptability [16,18]. Incorporating detailed cytological tumor grading into routine FNAC reporting provides vital prognostic guidance, facilitating optimal decision-making for neoadjuvant chemotherapy and tailored surgical interventions [19,20].

Discriminant analysis from earlier classical investigations demonstrated that nuclear features-particularly nuclear diameter, pleomorphism, and nucleoli-exhibit the closest correlation with histological grade, supporting the utility of scoring aspirates into high- and low-grade categories [21,22]. Traditional systems, such as Robinson’s schema, rely heavily on nuclear characteristics and general cellular discohesion [9]. While effective, conventional criteria often encounter diagnostic challenges in differentiating well-differentiated low-grade carcinomas from florid proliferative ductal hyperplasias or accurately stratifying intermediate-grade tumors [23,24]. By integrating cluster edge regularity and the relative preservation versus loss of bipolar myoepithelial cells, our nine-parameter approach captures critical biological hallmarks of stromal invasion and loss of tissue polarization [14,17].

A comparative evaluation of diagnostic performance reveals that while the Bethesda-based guidelines offer high sensitivity (reaching up to 98.4%), they often suffer from lower specificity (approximately 60%) in distinguishing atypical lesions [12]. Similarly, the IAC Yokohama system demonstrates a sensitivity range of 81.8% to 94.7% depending on the diagnostic scenario, with specificity peaking at 96.4% to 100% when only definitive malignant categories are considered [24,25]. The agreement observed between our cytological grading system and the Nottingham histological grade (kappa w = 0.908, 95% CI: 0.812-1.000) compares favorably with reported kappa statistics in the literature, which typically range from 0.65 to 0.85 [14,26,27]. Specifically, the 100% concordance observed in Grade III malignancies is of paramount clinical utility, as high-grade tumors reflect biologically aggressive phenotypes [20]. To further contextualize the present findings, Table 5 delineates a structured comparison between previous cytological grading systems and our improvised nine-parameter approach.

**Table 5:** Comparison of previous research frameworks with the present advanced nine-parameter cytomorphological grading system. N:C: nuclear:cytoplasmic; NCI: National Cancer Institute; MEC: myoepithelial cell; EC: epithelial cell; IAC: International Academy of Cytology

Study/framework	System evaluated	Parameters assessed	Diagnostic strengths	Key weaknesses/limitations	Comparison with present study
NCI Bethesda (1997) [12]	5-tier diagnostic risk categorization	General cellularity, architectural pattern, and background necrosis	Standardized uniform reporting terminology across institutions	Lacks specific numerical tumor grading; wide gray zone in indeterminate categories (low specificity ~60%)	The present system uses Bethesda as a preliminary filter, then applies numerical grading (9-27) specifically to Category 5/malignant lesions.
IAC Yokohama (2016) [5,6,13]	5-tier risk stratification system	Risk-of-malignancy categorization (insufficient, benign, atypical, suspicious, malignant)	Excellent general specificity (96.4%-100%) for definitive malignancy	Does not provide sub-tier cytological grading to predict histological Nottingham grade	The present system achieves equal specificity while adding a 3-tier cytological grading rubric that matches histology (kappa w = 0.908).
Hunt et al. (1990) & Fisher et al. (1981) [21,22]	Nuclear grading schemes	Nuclear size, nuclear pleomorphism, and presence/size of nucleoli	Strongly correlated with histological nuclear grade and ER receptor status	Ignored architectural cohesiveness, cluster margins, and background stromal cells/MECs	The present system expands nuclear assessment by incorporating cluster margin contours and bare MEC distribution.
Robinson et al. (1994) [8]	6-parameter cytological grading (scores 6-18)	Cell dissociation, cell size, uniformity, nucleoli, nuclear margin, and chromatin	Simplicity, wide adoption, good correlation in overt high-grade tumors	Tends to overestimate Grade I proliferative hyperplasias; moderate concordance in intermediate Grade II (56%-78%)	The 9-parameter system resolved 4 discordant Robinson cases and achieved higher overall concordance (92.0% vs. 78.0%).
Yu et al. (1998) [17]	Discohesion score (DS)	Proportion of intact clusters vs. loosely cohesive/single cells	Correlated tumor discohesion directly with axillary lymph node metastases in lesions < 3 cm	Did not incorporate a comprehensive multi-parameter nuclear scoring rubric	The present system adopts cluster cohesiveness and margin irregularity as core scored criteria (1-3 points each).
Sinha et al. (2009) [14]	Robinson's system vs. Bloom-Richardson	6 Robinson parameters on Pap smears correlated with Nottingham grade	Validated Robinson's utility in high grades (kappa = 0.829 for Grade III)	Fair-to-moderate agreement in Grade I and II lesions (kappa = 0.63-0.65)	The improvised system improves overall inter-method agreement to kappa w = 0.908 across all grades in this series.
Present study (2026)	Advanced 9-parameter system (scores 9-27)	Cluster cohesiveness, cluster margins, EC/MEC distribution, cell size, uniformity, N:C ratio, nuclear margins, chromatin, nucleoli	Kappa w = 0.908, chi square = 238.2 (p < 0.001); 100% concordance in Grade III; effectively resolves borderline Category 3/4 gray zones	Requires at least 6 intact cell clusters; mitotic count and tubular architecture cannot be directly evaluated on smears	Bridges the gap between low-complexity cytological scoring and final histological Elston-Ellis Nottingham grading.

​Despite the encouraging diagnostic performance, this study has several important limitations that must be acknowledged: (1) Single-center design and sample size: the sample size was modest (106 aspirates and 50 histological follow-up specimens) from a single academic institution. (2) Verification (workup) bias: histological confirmation was restricted to 50 mastectomy cases; benign and atypical cases without subsequent surgical excision were not subjected to full histological assessment, introducing partial verification bias and precluding full diagnostic accuracy evaluation across non-malignant cohorts. (3) Lack of reproducibility assessment: although smears were evaluated in consensus by cytopathologists blinded to surgical outcomes, formal independent multi-observer and intraobserver reproducibility assessments (such as multi-rater kappa testing) were not performed in this preliminary investigation. (4) Fundamental cytological constraints: fundamental cytological constraints prevent direct assessment of glandular/tubular architecture and mitotic counts, which are intrinsic components of the Elston-Ellis histological grade. (5) Absence of molecular correlates: the study did not correlate cytomorphological grades with breast cancer immunohistochemical subtypes (ER, PR, HER2/neu, and Ki-67 proliferation index), which govern modern systemic oncology and definitive neoadjuvant chemotherapy protocols. (6) Preparation method constraints: the system was evaluated using conventional manual smear preparations (Pap and Leishman-Giemsa) and was not tested on liquid-based cytology (LBC) platforms. (7) Requirement for external validation: the scoring thresholds were derived and evaluated within the same institutional cohort; prospective external validation across larger, multi-center datasets is necessary before recommending routine clinical implementation.

## Conclusions

The improvised nine-parameter cytomorphological grading system demonstrated strong concordance with Nottingham histological grades in this cohort of 50 surgically treated breast carcinomas, yielding a linear weighted kappa of kappa w = 0.908 (95% CI: 0.812-1.000) and showing improved intermediate-grade stratification compared to Robinson’s schema. While it provides an accessible, low-cost morphological risk-assessment tool for resource-constrained settings, definitive treatment planning requires core biopsy evaluation with biomarker profiling. Further prospective multi-center studies evaluating interobserver reproducibility and external validity are warranted before routine clinical adoption can be considered.
